# Enhanced Biocontrol of Cucumber Fusarium Wilt by Combined Application of New Antagonistic Bacteria *Bacillus amyloliquefaciens* B2 and Phenolic Acid-Degrading Fungus *Pleurotus ostreatus* P5

**DOI:** 10.3389/fmicb.2021.700142

**Published:** 2021-08-24

**Authors:** Hongwei Wang, Xiao-Yu Cai, Man Xu, Feng Tian

**Affiliations:** Nanjing Institute of Environmental Science, Ministry of Ecology and Environment of China, Nanjing, China

**Keywords:** biological control, *Fusarium oxysporum* f. sp. *cucumerinum* (FOC), phenolic acid, biodegradation, scanning electron microscopy

## Abstract

Continuous monoculture of cucumber (*Cucumis sativus* L.) typically leads to the frequent incidence of Fusarium wilt caused by *Fusarium oxysporum* f. sp. *cucumerinum* (FOC). As potent allelochemicals, phenolic acids are believed to be associated with soilborne diseases. This study aimed to investigate the effect of single or co-inoculation of antagonistic bacteria *Bacillus amyloliquefaciens* B2 and phenolic acid-degrading fungus *Pleurotus ostreatus* P5 on the suppression of cucumber Fusarium wilt. The strain B2 was identified as *B. amyloliquefaciens* based on biochemical, physiological, and 16*S* rDNA and *gyrB* gene sequence analyses. Strain B2 showed indole-3-acetic acid (IAA) and siderophore production and phosphate solubilization in *in vitro* assays. Scanning electron microscope (SEM) imaging showed the ability of strain B2 to adhere to the root surface of cucumber. *P. ostreatus* P5 could effectively degrade mixed phenolic acids as its sole source of carbon and energy for growth in liquid medium. In a pot experiment, four treatments were established as follows: (1) CK, uninoculated control; (2) B2, inoculation of strain B2; (3) P5, inoculation of strain P5; and (4) B2 + P5, co-inoculation of strain B2 and strain P5. At the end of the 60-day pot experiment, the B2, P5, and B2 + P5 treatments significantly reduced disease incidence by 48.1, 22.2, and 63.0%, respectively, compared to the CK treatment (*p* < 0.05). All three inoculation treatments significantly increased the growth of cucumber seedlings and suppressed the FOC population compared to the control (*p* < 0.05). High-performance liquid chromatography (HPLC) analysis showed that total phenolic acids were decreased by 18.9, 35.9, and 63.2% in the B2, P5, and B2 + P5 treatments, respectively. The results from this study suggest that combined application of *B. amyloliquefaciens* B2 and *P. ostreatus* P5 could be a promising strategy for suppressing Fusarium wilt and improving plant growth of cucumber seedlings under continuous cropping conditions.

## Introduction

Cucumber (*Cucumis sativus* L.) is an economically important vegetable crop worldwide, and Fusarium wilt of cucumber caused by *Fusarium oxysporum* f. sp. *cucumerinum* (FOC) has become the major limiting factor in the cucumber continuous cropping system (Ye et al., 2006; Raza et al., 2017). FOC is a soilborne pathogen that invades the vascular system of cucumber and eventually causes growth retardation, yellowing and necrosis of foliage, and death of the plant (Gordon, 2017). Traditional control strategies, including the use of fungicide, rotation, and resistant cultivar, have been suggested to control cucumber Fusarium wilt; however, these approaches are not environmentally friendly, economical, or reliable (Cao et al., 2011; Raza et al., 2017; Han et al., 2019). Biological control represents an attractive alternative method for protection of crops against Fusarium wilt. Many microbial species such as *Bacillus* spp., *Pseudomonas* spp., *Trichoderma* spp., *Streptomyces* spp., and *Acinetobacter* spp. have been shown to effectively control FOC (Raza et al., 2017; Kumar et al., 2020; Netzker et al., 2020). The primary mechanism probably involves secreting antimicrobial compounds, competing for biological niche and nutrients, and inducing plant resistance (Shafi et al., 2017; Netzker et al., 2020).

In addition to soilborne plant pathogens, autotoxic phenolic acids that are produced by plant leaching, root exudation, and residue decomposition tend to accumulate in continuous cropping soil and are typically considered to be involved in the pathogenicity caused by *Fusarium* spp. (Chen et al., 2011; Wu et al., 2015; Ferruz et al., 2016; Li et al., 2017; Tian et al., 2019; Wang et al., 2019; Jin et al., 2020). Autotoxic phenolic acids such as cinnamic acid cause oxidative damage in cucumber roots and predispose cucumber plants to infection by pathogens (Ye et al., 2004, 2006; Li et al., 2016). Additionally, in an *in vitro* experiment, *p*-hydroxybenzoic acid, ferulic acid, and cinnamic acid from the roots of watermelon stimulate *Fusarium oxysporum* f. sp. *niveum* spore germination, sporulation, and growth (Lv et al., 2018). In the rhizosphere of *Rehmannia glutinosa*, phenolic acids have also been found to induce the mycelial growth and toxin production of the soilborne pathogen *F. oxysporum* (Wu et al., 2015). Zhao et al. (2018) also reported that some phenolic acids stimulated the production of fusaric acid of *F. oxysporum* and thereby contributed to the incidence of root rot disease of ginseng. Therefore, reducing phenolic acid content in continuous cropping soil will likely alleviate crop Fusarium wilt (Zhou et al., 2020).

Recently, *Pleurotus ostreatus*, a member of the group of white rot fungi, has been studied due to its strong ability to degrade a diverse range of complex organic pollutants by extracellular lignin-mineralizing enzymes (i.e., laccases and peroxidases) and intracellular enzymatic complexes (e.g., cytochrome P450) (de Freitas et al., 2017; Brugnari et al., 2018; Mallak et al., 2020). Previous studies have demonstrated the laccase-mediated processes of biodegradation of phenolic acids in liquid medium and natural soil (Xie and Dai, 2015; Xie et al., 2016). Because *P. ostreatus* has a strong laccase-secreting ability (Brugnari et al., 2018), it is likely to be a promising agent for phenolic acid removal.

Combined use of two or more biocontrol candidates, a combination of bacterial–bacterial, bacterial–fungal, or fungal–fungal isolates, in managing various important plant diseases has been used for many years (Awasthi et al., 2011; Yobo et al., 2011; Zaim et al., 2018; Jangir et al., 2019; Hansen et al., 2020). These methods also showed better efficacy compared to using a single beneficial microbe (Kohler et al., 2007; Awasthi et al., 2011; Jangir et al., 2019). Duijff et al. (1998) showed that a synergistic effect can be obtained in controlling the Fusarium wilt of tomato by combining a *Pseudomonas fluorescens* WCS417r with a non-pathogenic *Fusarium oxysporum* Fo47. Additionally, combined inoculation of plant growth-promoting rhizobacteria (PGPR) and mycorrhizae was more efficient in the control of plant fungal pathogens in common beans than a single inoculation (Mohamed et al., 2019). Several studies have demonstrated the possibility of suppressing cucumber Fusarium wilt in continuous cropping systems using single antagonistic (Cao et al., 2011; Han et al., 2019) or phenolic acid-degrading (Chen et al., 2011) microbes; however, combined application of these different functional microbes has rarely been studied. Co-inoculations of antagonistic agent and phenolic acid-degrading microbe might provide a higher level of protection and wider range of effectiveness by activating several different mechanisms.

In this study, we identify an antagonistic strain B2 isolated from rhizosphere soil of continuously cropped cucumber and describe its plant-beneficial traits. Second, the ability of *P. ostreatus* P5 to degrade the phenolic acids is investigated. Last, the potentials of strains B2 and P5, alone or in combination, are evaluated to suppress cucumber Fusarium wilt in a pot experiment.

## Materials and Methods

### Strains

An antagonistic microorganism was isolated from the rhizosphere soil of a healthy cucumber plant in cucumber-growing areas of the Nanjing Institute of Vegetable Science (118°46.615’E, 31°43.195’N) in Nanjing, Jiangsu Province, China. The soil suspension was serially diluted 10-fold, spread on Luria–Bertani (LB) medium, and incubated at 30°C for 3 days. Purified colonies were screened for their antagonism to fungal pathogen FOC on plates using the dual culture method (Rocha et al., 2017). The bacterial strain showing the strongest antifungal effect was coded as B2 and selected for further studies.

*Pleurotus ostreatus* strain P5 was isolated from spent *P. ostreatus* substrate (Wang et al., 2020). The fungal isolate was maintained on potato dextrose agar (PDA) slants at 4°C.

The pathogen used throughout this study was FOC [Agricultural Culture Collection of China (ACCC) No. 30220], which was provided by the Institute of Plant Protection, Chinese Academy of Agricultural Sciences. FOC was stored on PDA slants at 4°C.

### Soil

**FIGURE 1:**
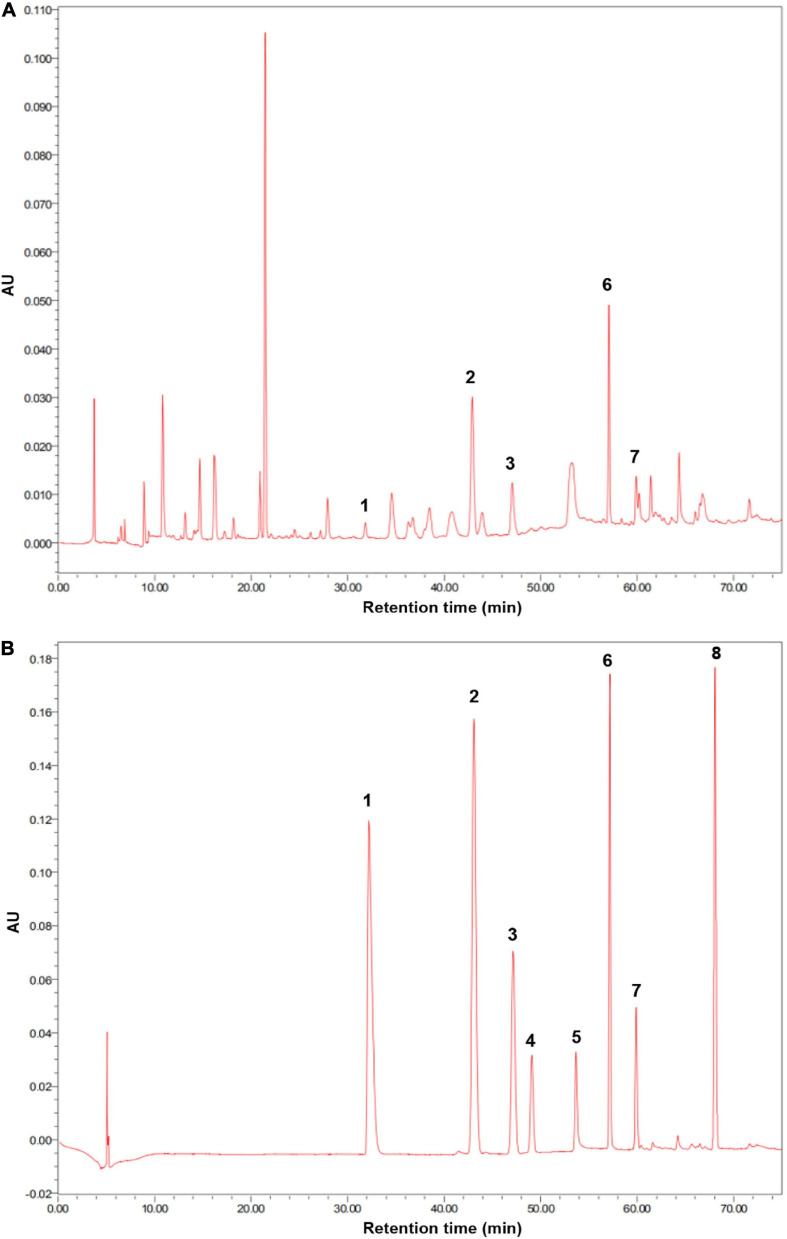
**(A)** High-performance liquid chromatography (HPLC) chromatogram of extracts obtained from cucumber continuous cropping soil was used in this experiment. **(B)** HPLC chromatogram of standard phenolic acids. (1) *p*-coumaric acid; (2) *p*-hydroxybenzoic acid; (3) vanillic acid; (4) syringic acid; (5) vanillin; (6) ferulic acid; (7), benzoic acid; (8) cinnamic acid.

The soil used in this experiment was collected from the surface layer of soil (0–15 cm) from a greenhouse at the Nanjing Institute of Vegetable Science. The soil at this site is classified as yellow-brown earth based on the Chinese soil classification and as Hapludalf, Alfisols based on the USDA’s soil classification (Wang et al., 2017). The studied greenhouse has continuously produced cucumbers for over 10 years. The collected soil was homogenized, passed through a 2-mm sieve at field moisture, and stored at 4°C until being used. The soil pH was 6.1; the organic carbon content was 11.3 g kg^–1^; and the contents of total nitrogen, total phosphorus, and total potassium were 1.73 g kg^–1^, 1.64 g kg^–1^, and 17.6 g kg^–1^, respectively. The soil contained *p*-hydroxybenzoic acid, vanillic acid, ferulic acid, *p*-coumaric acid, and benzoic acid at concentrations of 84.7, 36.4, 59.2, 21.6, and 28.5 μg/g, respectively (Figure 1). Soil phenolic acid extraction and detection were performed as follows.

Phenolic acids were extracted and detected by the method described by Chen et al. (2011). Briefly, 50 g of soil was extracted with 50 ml of 1 M NaOH and kept at 25°C for 24 h in the dark. Samples were then shaken vigorously for 30 min and centrifuged at 10,000 r min^–1^ for 10 min. The 12 M HCl was used to adjust the pH of the supernatant to 2.5. The solutions were extracted three times with an equal volume of diethyl ether and dried in a rotary evaporator. The solvent was dissolved in 1 ml acetonitrile and filtered through 0.45-μm fiber filtration film. Extracts were analyzed using a Waters e2695 high-performance liquid chromatography (HPLC) (Milford, MA, United States), containing a YMC ODS-A column (4.6 mm × 250 mm, 5 μm). The standard phenolic acids (Shanghai Aladdin Biochemical Technology Co., Ltd.) used for HPLC were *p*-hydroxybenzoic acid, *p*-coumaric acid, ferulic acid, cinnamic acid, syringic acid, vanillic acid, vanillin, and benzoic acid. The phenolic acids in the extracts were separated using a gradient elution of solvent A acetic water (0.2%) and solvent B acetonitrile at 254 nm with a flow rate of 0.8 ml min^–1^. The gradient elusion program was as follows: for 0–27 min, 0–10% solvent B; for 27–42 min, 10–15% solvent B; for 42–50 min, 15–30% solvent B; for 50–60 min, 30–50% solvent B; and for 60–70 min, 50–100% solvent B. The concentrations of phenolic acids were calculated from the standard curves. The presence of phenolic acid was confirmed by an ESI mass spectrometer AB SCIEX Triple Quad^TM^ 4500 using positive ion mode (AB SCIEX, Framingham, MA, United States).

### Identification of *B. amyloliquefaciens* B2

The traditional physiological and biochemical characteristics of strain B2 were identified based on Bergey’s Manual of Systematic Bacteriology. Strain B2 was further identified through the analysis of its 16S rDNA and *gyrB* gene sequences. Briefly, the genomic DNA of the strain B2 was extracted using the bacterial DNA extraction kit (Omega, Germany) and stored at –20°C. The 16S rDNA was amplified with the bacterial universal primers 27F (5′-AGAGTTTGATCCTGGCTCAG-3′) and 1492R (5′-GGTTACCTTGTTACGACTT-3′) (Eden et al., 1991), and the *gyrB* gene was amplified with the specific primers UP1 (5′-GAAGTCATCATGACCGTTCTGCAYGCNGGNGGNAARTTY GA-3′) and UP2r (5′-AGCAGGGTACGGATGTGCGAGCCRT CNACRTCNGCRTCNGTCAT-3′) (Yamamoto and Harayama, 1995). The 20-μl PCR mixture contained 2 μl dNTP (2 mM), 2 μl MgCl_2_ (25 mM), 1.0 μl of each primer (10 mM), 2.0 μl PCR buffer (10×), 1.0 μl template DNA, 0.2 μl Taq DNA polymerase (5 U), and 10.8 μl double-distilled (dd) H_2_O. The thermocycling procedure involved an initial denaturation at 95°C for 3 min, followed by 35 cycles at 95°C for 1 min, 50°C for 45 s, 72°C for 2 min, and a final extension at 72°C for 10 min. The PCR products were then purified and sequenced by Majorbio Bio-pharm Technology Co., Ltd. (Shanghai, China). A sequence similarity analysis was performed using the NCBI BLAST program^1^, and the phylogenetic tree was constructed by the neighbor-joining (NJ) method using MEGA-X.

### Plant-Beneficial Traits of *B. amyloliquefaciens* B2

#### Plant Growth-Promoting Traits

Indole-3-acetic acid (IAA) production was checked by inoculating strain B2 into 50 ml of LB broth amended with 5 mM L-tryptophan for 48 h in the dark at 28°C (Patten and Glick, 2002). IAA production was quantitated spectrophotometrically at 595 nm using Salkowski’s reagent (4.5 g of FeCl_3_ per L in 10.8 M H_2_SO_4_). Phosphate solubilization activity was tested on Pikovaskaya’s agar medium containing 2% tricalcium phosphate (Kucey, 1987). The appearance of a clear halo zone around bacterial colonies after incubation for 7 days at 28°C was indicative of phosphate solubilization. Siderophore production was detected by the formation of orange halos around bacterial colonies on Chrome Azural S (CAS) agar plates after 3 days’ incubation at 28°C (Schwyn and Neilands, 1987). Nitrogen fixation ability was tested in nitrogen-free Ashby medium based on the process described by Kızılkaya (2008).

#### Biofilm Assay

Biofilm formation of strain B2 was determined quantitatively through the crystal violet assay described by Peeters et al. (2008). The biofilm was quantified by measuring the OD_590_ of the crystal violet–ethanol solution with a microplate spectrophotometer (BioTek Instruments Inc., United States).

#### Colonization Ability on Root Surface

Strain B2 was grown overnight in 100 ml of LB medium at 30°C on a rotary shaker. Bacterial cells were collected by centrifugation and suspended in LB medium to obtain a final inoculum density of 1 × 10^8^ CFU ml^–1^. The cucumber seeds were surface sterilized by soaking in 70% ethanol for 2 min followed by treatment with 2% sodium hypochlorite for 5 min. The seeds were then washed four times with sterile distilled water. Seed sterility was ascertained by incubating the seeds on LB agar plates at 30°C for 4 days and checking for the absence of bacterial contamination. The seeds were then kept for 2 h in the bacterial solution (1 × 10^8^ CFU ml^–1^) then briefly rinsed in sterile distilled water to remove non-adherent bacteria. The inoculated seeds were plated on Murashige and Skoog (MS) agar medium and incubated in a vertical position under controlled environmental conditions (22°C; 16 h/8 h light/dark) for germination and root elongation. Seven-day-old fresh roots of seedlings were collected directly from the plates and washed briefly in sterile water in preparation for scanning electron microscope (SEM) imaging. Roots were cut into 5-mm lengths and fixed in a 3% glutaraldehyde buffered with 0.1 M phosphate buffer (pH 7.0) for 24 h at 4°C. Root samples were then thoroughly rinsed in 0.1 M phosphate buffer (pH 7.0) and dehydrated at 25°C using a graded ethanol series (25, 50, 75, 85, and 100% ethanol). Last, the samples were dried with a critical point dryer, sputter-coated with platinum, and viewed in SEM (Jeol, Tokyo, Japan).

Single strain B2 was also observed using SEM. Briefly, after incubation in LB for 48 h at 30°C, strain B2 was collected by centrifugation. After washing three times with phosphate buffer, strain B2 was fixed with 3% glutaraldehyde in phosphate buffer at 4°C for 24 h. After washing three times with phosphate buffer, the samples were dehydrated using a graded series of ethanol solutions (25, 50, 75, 85, and 100% ethanol). They were then dried, sputter-coated, and viewed with the SEM.

### Identification of Optimal Concentration for *P. ostreatus* P5 Degradation

To study the effects of different initial concentrations of mixture of phenolic acids [*p*-hydroxybenzoic acid, vanillic acid, ferulic acid, *p*-coumaric acid, benzoic acid (1/1/1/1/1, w/w)] on degradation, 2-ml inocula containing 1.2 mg L^–1^ of mycelia were added to 50-ml mineral salt medium (MSM; KCl 0.5 g, K_2_HPO_4_ 1 g, KNO_3_ 2 g, MgSO_4_ 0.5 g, FeSO_4_ 0.01 g, and distilled water 1,000 ml—all autoclaved at 121°C for 30 min) with 100–1,000 mg L^–1^ mixture of phenolic acids as the sole carbon and energy source. All treatments were incubated at 28°C and 180 rpm for 96 h. Each treatment had three replicates, and no fungal inoculation was used as a control. The fungal mycelia in the culture were filtered and washed with acetonitrile three times and dried at 80°C until having a constant weight. Phenolic acids were extracted three times with diethyl ether, and the combined extracts were dried *via* rotary evaporator. The residues were dissolved in 1 ml acetonitrile, and the amounts of phenolic acids in the acetonitrile were determined with HPLC, as described in Section Soil.

### Phenolic Acid Degradation in Liquid Culture by *P. ostreatus* P5

The mixture of phenolic acid (400 mg L^–1^; *p*-hydroxybenzoic acid 80 mg L^–1^, vanillic acid 80 mg L^–1^, ferulic acid 80 mg L^–1^, *p*-coumaric acid 80 mg L^–1^, benzoic acid 80 mg L^–1^) was used to evaluate the degradation ability of strain P5 based on the fungal biomass, and the degradation rate was relatively high at this concentration. The inoculum size and culture conditions were consistent with those mentioned in Section Identification of Optimal Concentration for *P. ostreatus* P5 Degradation, and the flasks without strain P5 inoculation were used as control.

Kinetics of the phenolic acid degradation in liquid culture were determined by harvesting samples after 0, 12, 24, 36, 48, 60, 72, 84, and 96 h and freezing the samples at –80°C for later analysis. The fungal mycelia biomass and residual phenolic acid concentrations were detected as described above.

### Greenhouse Pot Experiment

The strain B2 was cultivated in LB medium as described above. Cells were harvested by centrifugation, and cell pellets were resuspended twice with sterile distilled water. The cell concentration of strain B2 in the suspension was adjusted to 1 × 10^8^ CFU ml^–1^ by diluting it with sterile distilled water. *P. ostreatus* strain P5 was grown for 5 days in PDA liquid medium at 28°C (180 rpm). The culture was centrifuged, and the obtained fungal mycelia were washed twice with sterile distilled water and were used as fungal inocula. Cucumber seeds (*Cucumis sativus* L., JinChun-No. 4, Tianjin Cucumber Research Centre) were surface sterilized with ethanol and 2% sodium hypochlorite (2 and 5 min, respectively), rinsed four times in sterilized distilled water, and then germinated in 90-mm glass Petri plates covered with moist filter paper in the dark at 30°C for 36 h.

After germination, the seeds were sown into nursery cups containing 300 g sterilized (121°C, 30 min) nursery soil. Plant seedlings (two true-leaf stage) were transplanted into plastic pots (15 cm diameter, 20 cm height) with 2 kg of soil. Three days before transplanting, the soil was inoculated with spores of FOC at concentrations of 1 × 10^4^ CFU g^–1^ of soil. The pot experiment was designed in a randomized complete block design with four replications. The various treatments were as follows: (1) CK, without any microbial treatment; (2) B2, inoculation with strain B2; (3) P5, inoculation with strain P5; and (4) B2 + P5, co-inoculation with strain B2 and strain P5. One day before transplanting, wet fungal mycelia (20.0 g wet weight equivalent to 2.14 g dry fungal weight) were suspended in 50 ml water and mixed with 2 kg of soil. After transplanting, 10 ml of a water solution with strain B2 (10^8^ CFU ml^–1^) was added around the root zone of the plant seedlings in the relevant treatment using a syringe. To maintain uniformity of nutrient supply in the four treatments, non-bacterial treatments received strain B2 inocula that had been autoclaved (121°C, 30 min), and non-fungal treatments also received strain P5 inocula that had been autoclaved (121°C, 30 min). Each treatment had four blocks with 12 pots in each block. One seedling was grown in each plastic pot. The pot experiment was conducted in a greenhouse located at the Nanjing Institute of Environmental Science, Nanjing, China. The temperature ranged from 21 to 28°C, and the relative humidity ranged from 64 to 83%.

After 60 days of transplanting, the cucumber seedlings were gently removed from the pot and shaken lightly to remove the soil loosely attached to the plant roots. The soil tightly adhering to the roots was collected and considered rhizosphere soil. The rhizosphere soil samples were divided into two subsamples: one was stored at –80°C for molecular studies and the other was stored at –20°C for phenolic acid analysis. Seedling infection by FOC was monitored daily, and the cumulative number of infected seedlings was also recorded. Disease incidence (DI) was defined as the percentage of infected seedlings over the total number of seedlings in each block and was assessed when the disease symptoms appeared (>20% of leaves wilted) (Cao et al., 2011). After harvesting, the plant height, root length, plant dry weight, and root dry weight were also measured.

### Real-Time PCR Assay

Total soil DNA was extracted from 0.25 g of rhizosphere soil using the UltraClean Soil DNA Isolation Kit (Mo Bio Laboratories Inc., United States). The FOC-specific SCAR primer FocF3 5′-AAACGAGCCCGCTATTTGAG-3′ and FocR7 5′-TATTTCCTCCACATTGCCATG-3′ designed by Lievens et al. (2007) was used in a real-time PCR assay. The real-time PCR conditions were previously described by Cao et al. (2011). Real-time PCR was run in an ABI 7500 instrument (Applied Biosystems, United States). For quantification, standard curves were created with a 10-fold dilution series of plasmids (pMD-19T vector, Takara Bio) containing the PCR products from the amplification of FOC DNA with the primer FocF3/FocR7. All real-time PCRs were performed in triplicate, and ddH_2_O was used as a negative control to replace the template.

### Soil Phenolic Acids

The extraction and determination of soil phenolic acids were performed as described above.

### Statistical Analysis

All data were analyzed using SPSS 17.0 statistical software. Statistical significance was evaluated at the 95% level with Duncan’s test (*p* < 0.05). The data from the pot experiment followed a normal distribution (Shapiro–Wilk test) except for abundance of FOC. Pearson correlations were used for disease incidence and phenolic acid content with normal distribution, and Spearman rank correlations were used for disease incidence and FOC (R software Version 3.2.2).

## Results

### Identification of *B. amyloliquefaciens* B2

**FIGURE 2:**
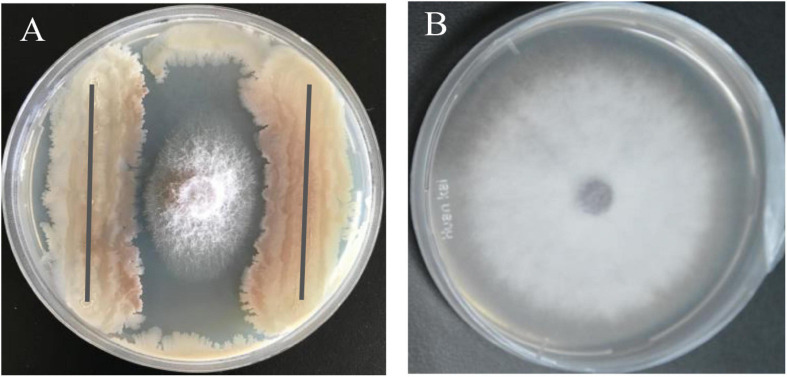
Antagonism of *B. amyloliquefaciens* B2 against plant pathogen *F. oxysporum* f. sp. *cucumerinum* (FOC). **(A)** Antagonistic effects of strain B2 against FOC. **(B)** FOC grown on potato dextrose agar (PDA) plate as control.

**FIGURE 3:**
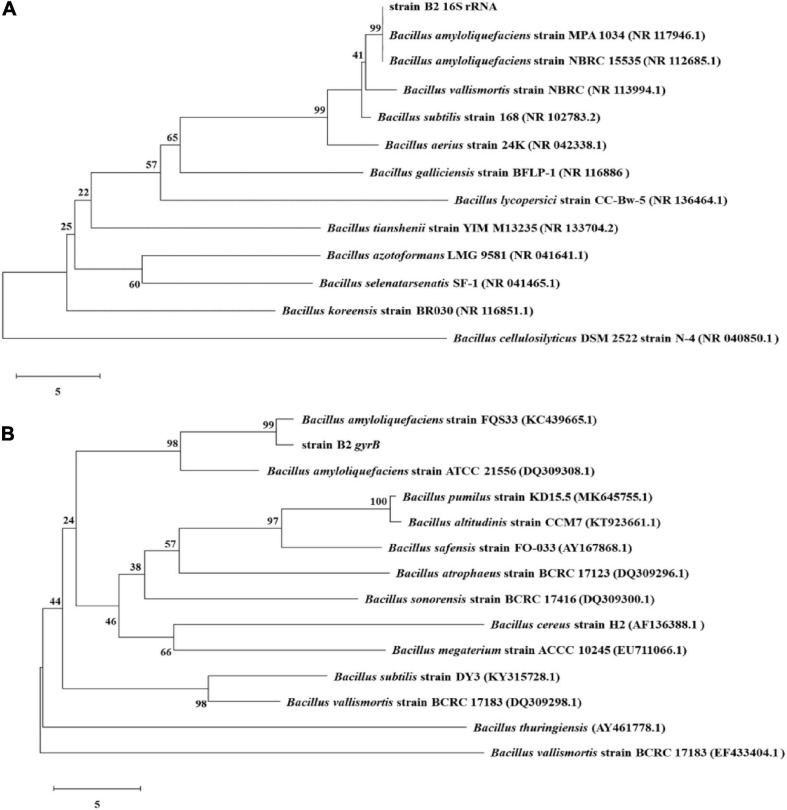
Phylogenetic trees constructed based on 16S rRNA **(A)** and *gyrB* **(B)** gene sequences of *B. amyloliquefaciens* B2 and other *Bacillus* strains. Bootstrap values (%) shown at the branches were calculated from 1,000 replications.

The bacterial strain B2, an antagonist against FOC (Figure 2), was obtained from the rhizosphere soil of the cucumber. Physiological and biochemical characteristics are shown in Table 1. From these results, strain B2 was affiliated to the genus *Bacillus*. To further identify strain B2, 16S rDNA and *gyrB* genes were analyzed by sequencing. The 16S rDNA sequence of the strain B2 (GenBank accession number: MW308308) shared 99.8% identity with the 16S rDNA sequences of *B*. *amyloliquefaciens* (NR117946.1). The phylogenetic analysis of 16S rDNA sequences clearly showed that the strain B2 and *B*. *amyloliquefaciens* were clustered together (Figure 3A). Additionally, its *gyrB* gene sequence (GenBank accession number: MW316056) shared 99.8% identity with the *gyrB* gene sequences of *B*. *amyloliquefaciens* (KC439665.1). The phylogenetic analysis of *gyrB* gene sequences showed that the strain B2 and *B*. *amyloliquefaciens* were clustered together (Figure 3B). Thus, based on physiological and biochemical testing, and 16S rDNA and *gyrB* gene sequences analyses, strain B2 was identified as *B*. *amyloliquefaciens*.

**TABLE 1:** Biochemical and physiological characteristics of *B. amyloliquefaciens* B2.

**Item**	**Results**	**Item**	**Results**
Gram test	+	Catalase activity	+
MR	–	Carbohydrate utilization	
VP	+	Sucrose	+
Gelatin liquefaction	+	Maltose	+
Starch hydrolysis	+	Glucose	+
Tyrosine hydrolysis	–	Mannitol	+
Nitrate reduction	+	Xylose	+
H_2_S production	–	*L*-Arabinose	+
Oxidase activity	–		

*“+” Represents a positive reaction; “–” represents a negative reaction.*

### Plant-Beneficial Traits of *B. amyloliquefaciens* B2

**FIGURE 4:**
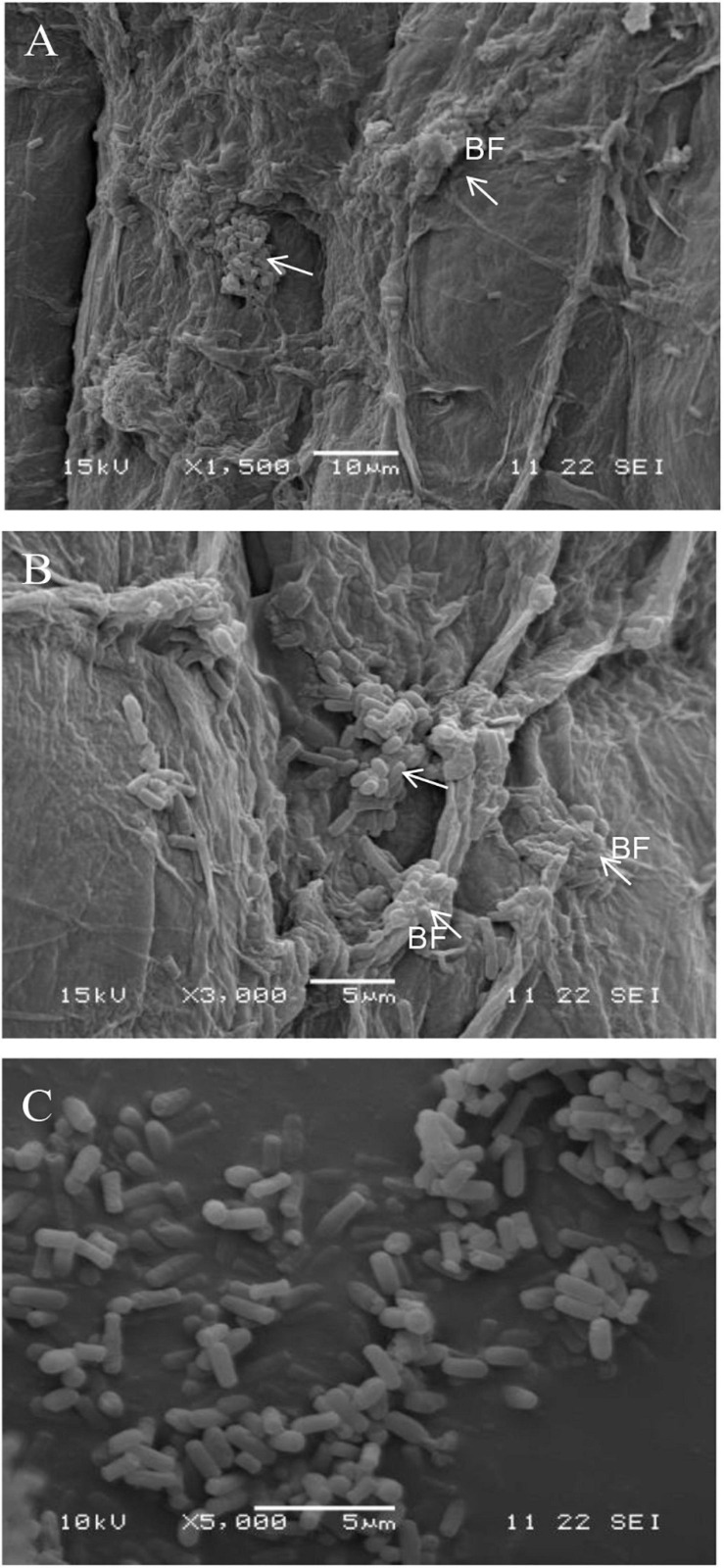
Scanning electron micrograph (SEM) showing the surface colonization of cucumber root by strain B2 at × 1,500 **(A)** and × 3,000 **(B)** and strain B2 cells **(C)**. *White arrow*, bacteria; *BF*, biofilm.

Strain B2 was tested for the presence of a number of plant-beneficial traits. The results of the characterization analysis showed that strain B2 had the ability to secret IAA (Table 2). Moreover, strain B2 was found to be positive for phosphate solubilization, siderophore production, and biofilm formation (Table 2). The root surfaces of cucumber seedlings that were inoculated with strain B2 were examined using scanning electron microscopy (SEM) (Figure 4). Bacterial cells were easily visible on the root surface and strongly adhered to the root surface because the sample preparation for SEM analysis requires multiple treatments and exhaustive washes. High-resolution images also showed bacteria-forming biofilm structures on the root surfaces (Figure 4).

**TABLE 2:** Beneficial traits of *B. amyloliquefaciens* B2.

**Item**	**Results**
IAA production (μg/ml)	42.7 ± 0.51
Siderophore	+
Phosphate solubilization	+
N_2_-fixation	–
Biofilm formation (OD590)	1.73 ± 0.19

*“+” Represents a positive reaction; “–” represents a negative reaction.*

### Phenolic Acid Degradation by *P. ostreatus* P5 in the Liquid Medium

**FIGURE 5:**
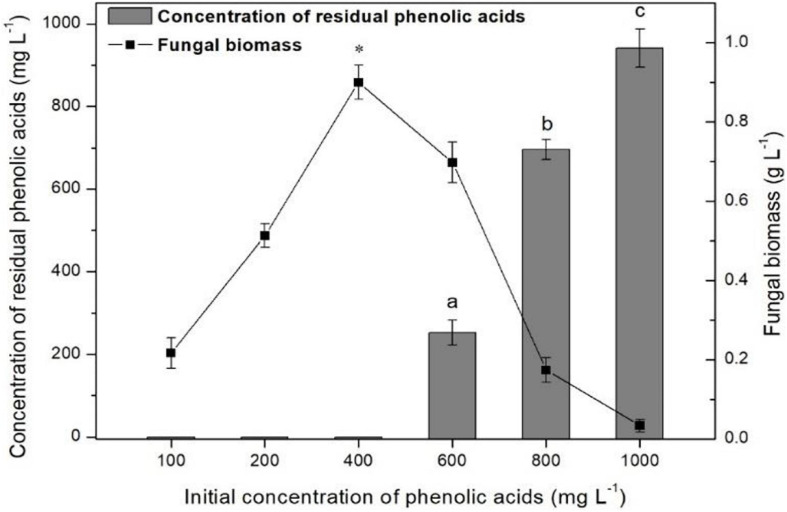
Detection of the optimum concentration of mixture of phenolic acids for *P. ostreatus* P5 degradation. Data are presented as the means of three independent replicates with standard deviation bars. Data in columns marked by different letters are significantly different (*p* < 0.05). “*” Indicates a significant difference from the other points (*p* < 0.05).

The ratios of mixture phenolic acid degradation and fungal biomass in different concentration cultures after 96 h of cultivation are shown in Figure 5. When the initial concentration was 400 mg L^–1^ or lower, 100% of the mixture of phenolic acids was degraded. However, if the concentration was greater than 400 mg L^–1^, the ratio of the mixture of phenolic acid degradation significantly decreased and only 5.9% could be removed at the concentration of 1,000 mg L^–1^. Moreover, the highest mycelial biomass was also observed at 400 mg L^–1^. Thus, 400 mg L^–1^ was chosen for the following degradation experiments.

**FIGURE 6:**
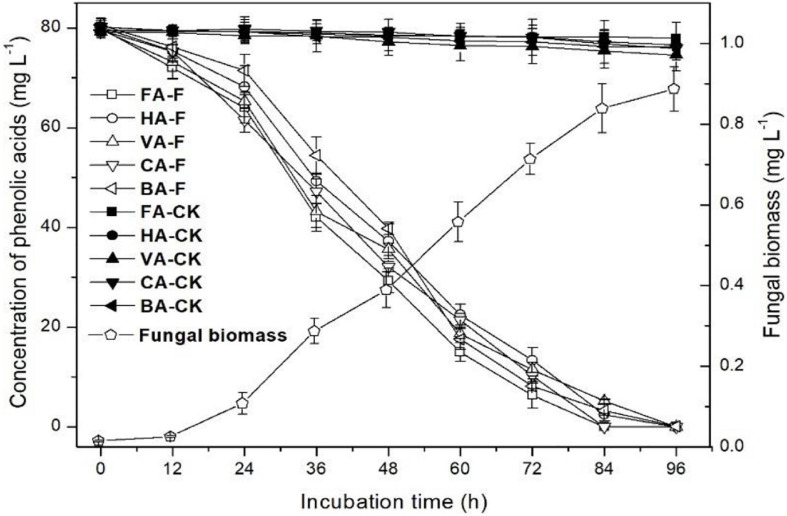
Degradation efficiency of mixture of phenolic acids by *P. ostreatus* P5. HA, *p*-hydroxybenzoic acid; VA, vanillic acid; FA, ferulic acid; CA, *p*-coumaric acid; BA, benzoic acid.

The kinetics of the mixture of five phenolic acid degradation and strain P5 growth in liquid medium are shown in Figure 6. In the control, natural degradation efficiencies of all five phenolic acids at 96 h of incubation were 2.7, 4.4, 7.2, 5.0, and 4.5% for ferulic acid, *p*-hydroxybenzoic acid, vanillic acid, cinnamic acid, and benzoic acid, respectively. After 84 h of incubation, ferulic acid and *p*-coumaric acid could not be detected in the strain P5-inoculated culture, and only 3.1% *p*-hydroxybenzoic acid, 6.5% vanillic acid, and 4.7% benzoic acid were left in the strain P5-inoculated culture. After 96 h of incubation, all phenolic acids added in the strain P5-inoculated culture were completely degraded, and the dry weight of strain P5 mycelia increased to 0.89 g dry weight per liter culture.

### Fusarium Wilt Incidents and Plant Seedling Growth

**FIGURE 7:**
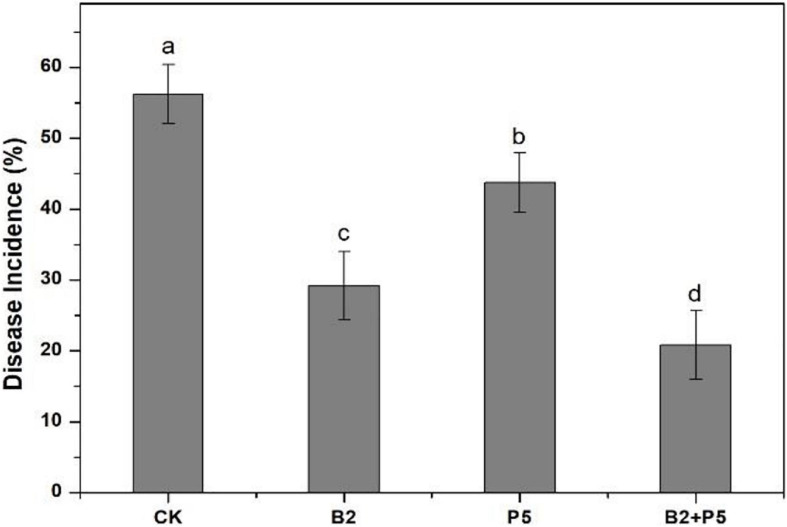
Effect of different treatments on the disease incidence of Fusarium wilt of cucumber seedlings at 60 days after transplanting. Treatments: CK, without any microbial treatment; B2, inoculation with *Bacillus amyloliquefaciens* B2; P5, inoculation with *Pleurotus ostreatus* P5; B2 + P5, co-inoculation with *B. amyloliquefaciens* B2 and *P. ostreatus* P5. Different letters above each bar indicate significant differences (*p* < 0.05; Duncan’s test) among treatments.

In the greenhouse pot experiment, the highest disease incidence (56.3%) occurred in the CK treatment (Figure 7). The application of strains B2 and P5 significantly decreased the disease incidence by 48.1 and 22.2%, respectively, compared to the CK treatment. However, the combined treatment B2 + P5 showed the best control efficacy of Fusarium wilt by 63.0% (Figure 7).

The results showed that application of strain B2 or P5 alone was effective for growth promotion in cucumber plants (Table 3). Additionally, the combined treatment B2 + P5 showed significantly stronger growth promotion effect compared to B2 and P5 treatments (Table 3). Plant dry weight, plant height, root length, and root dry weight with the B2 + P5 treatment increased by 64.6, 26.4, 37.6, and 53.8% compared to the control, respectively.

**TABLE 3:** Effects of four treatments on cucumber plant growth (60 days after transplantation).

**Treatment**	**Plant dry weight (g)**	**Plant height (cm)**	**Root length (cm)**	**Root dry weight (g)**
CK	8.2 ± 0.4^*a*^	91 ± 6.5^*a*^	14.1 ± 0.4^*a*^	1.3 ± 0.09^*a*^
B2	11.9 ± 0.8^*c*^	104 ± 4.3^*b*^	17.6 ± 0.7^*c*^	1.8 ± 0.14^*c*^
P5	10.4 ± 0.6^*b*^	101 ± 4.9^*ab*^	16.2 ± 0.4^*b*^	1.5 ± 0.11^*b*^
B2 + P5	13.5 ± 0.5^*d*^	115 ± 8.1^*c*^	19.4 ± 0.8^*d*^	2.0 ± 0.19^*c*^

*Treatments: CK, without any microbial treatment; B2, inoculation with *Bacillus amyloliquefaciens* B2; P5, inoculation with *Pleurotus ostreatus* P5; B2 + P5, co-inoculation with *B. amyloliquefaciens* B2 and *P. ostreatus* P5. Mean values and standard deviations are listed. Different letters in a column indicate significant differences (*p* < 0.05; Duncan’s test) among treatments.*

### Pathogen *F. oxysporum* f. sp. *cucumerinum* Detection in the Rhizosphere

**FIGURE 8:**
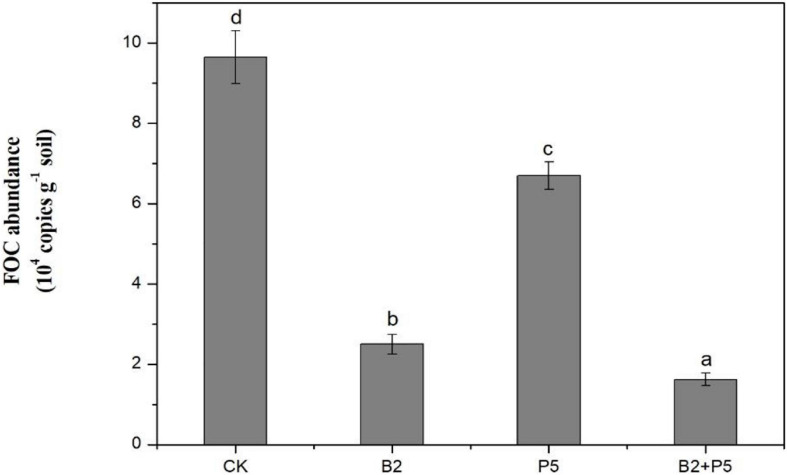
Effect of different treatments on the population of *F. oxysporum* f. sp. *cucumerinum* (FOC) in rhizosphere soil at 60 days after transplanting. Treatments: CK, without any microbial treatment; B2, inoculation with *Bacillus amyloliquefaciens* B2; P5, inoculation with *Pleurotus ostreatus* P5; B2 + P5, co-inoculation with *B. amyloliquefaciens* B2 and *P. ostreatus* P5. Different letters above each bar indicate significant differences (*p* < 0.05; Duncan’s test) among treatments.

A real-time PCR assay was used to determine the population of FOC in rhizosphere soil at the end of the experiments. The results indicated that the application of strain B2 or P5 alone significantly suppressed the population of FOC in rhizosphere soils, which was reduced by 74.0 and 30.5% compared to the CK treatment, respectively (Figure 8). The B2 + P5 treatment showed the strongest inhibitory effect on FOC population, which was only 1.62 × 10^4^ copies g^–1^ soil in the B2 + P5 treatment, corresponding to 83.2% reduction compared to control treatment (Figure 8).

### Soil Allelochemicals

All inoculation treatments reduced the contents of phenolic acids in the soil (Table 4). Compared to the CK treatment, the contents of total phenolic acids were significantly (*p* < 0.05) decreased by 18.9, 35.9, and 63.2% in B2, P5, and B2 + P5 treatments, respectively (Table 4).

**TABLE 4:** Phenolic acids in the soils of four treatments after harvesting the cucumber plant.

**Treatment**	**Concentration (μg g^–1^)**					
	**HA**	**VA**	**FA**	**CA**	**BA**	**Total**
CK	78.3 ± 3.4^*d*^	36.7 ± 3.2^*d*^	53.6 ± 2.1^*d*^	19.7 ± 0.9^*d*^	25.2 ± 1.8^*d*^	213.6 ± 8.6^*d*^
B2	58.9 ± 4.7^*c*^	30.9 ± 1.3^*c*^	45.1 ± 2.7^*c*^	16.2 ± 1.3^*c*^	22.1 ± 1.2^*c*^	173.1 ± 5.8^*c*^
P5	46.0 ± 3.3^*b*^	25.2 ± 2.3^*b*^	34.4 ± 2.0^*b*^	12.8 ± 1.3^*b*^	18.6 ± 1.1^*b*^	136.9 ± 6.7^*b*^
B2 + P5	25.4 ± 3.7^*a*^	19.6 ± 1.0^*a*^	15.4 ± 1.6^*a*^	7.2 ± 0.8^*a*^	11.0 ± 1.3^*a*^	78.5 ± 3.5^*a*^

*HA, *p*-hydroxybenzoic acid; VA, vanillic acid; FA, ferulic acid; CA, *p*-coumaric acid; BA, benzoic acid. Mean values and standard deviations are listed. Different letters in a column indicate significant differences (*p* < 0.05; Duncan’s test) among treatments.*

### Relationship Between Fusarium Wilt Disease Incidence and Soil *F. oxysporum* f. sp. *cucumerinum* and Phenolic Acids

**FIGURE 9:**
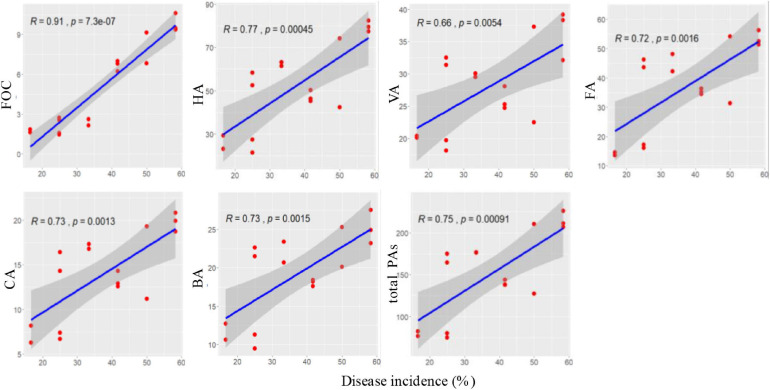
Correlations between Fusarium wilt disease incidence and soil FOC and phenolic acids at 60 days after transplanting.

Correlation coefficients were calculated between the disease incidence and abundance of FOC and content of phenolic acids at 60 days after transplanting. Disease incidence was significantly positively correlated with FOC and contents of *p*-hydroxybenzoic acid, vanillic acid, ferulic acid, *p*-coumaric acid, benzoic acid, and total phenolic acids (*p* < 0.05; Figure 9).

## Discussion

In this study, an antagonistic *B. amyloliquefaciens* B2 was isolated from rhizosphere soil sample of cucumber plants. The physiological and biochemical characteristics and phylogenetic analyses of the 16S rDNA and *gyrB* gene sequences confirmed that strain B2 was *B. amyloliquefaciens*. The isolation of *Bacillus* species from various crops’ rhizosphere soil has been extensively studied (Abdallah et al., 2018). Strain B2 produced significant amounts of IAA (42.7 μg ml^–1^), which might enhance root growth and enable the plant to uptake more nutrients from soil. Significant amounts of *in vitro* IAA production by *B. amyloliquefaciens* have also been documented by Abdallah et al. (2018) and Daraz et al. (2020). The production of siderophores by strain B2 might play an important role in promoting plant growth by providing Fe to the plant and by limiting the Fe availability to pathogens. Yu et al. (2011) also found that the *Bacillus subtilis* CAS15 produced a siderophore that had a biocontrol effect against Fusarium wilt and improved pepper growth. In this study, strain B2 was found to be a good solubilizer of phosphate. Phosphate solubilization has been identified and characterized previously from several *Bacillus* species, such as *B. amyloliquefaciens* (Abdallah et al., 2018), *B. subtilis* (Ahmad et al., 2017), and *B. pumilus* (Ansari et al., 2019).

Root colonization by the introduced rhizobacteria is necessary for the biocontrol agent to successfully establish efficient protection. SEM observations showed effective root colonization as the strain B2 developed a biofilm over the root surface. By adhering to plant roots, the bacteria will be able to exploit various compounds in root exudates such as sugar, amino acid, organic acid, and vitamin for their survival (Morris and Monier, 2003). The ability of strain B2 to form a biofilm is in line with previous results (Abdallah et al., 2018). Biofilm associated with the plant roots has been found to be beneficial for biocontrol and plant growth, as discussed in detail by Morris and Monier (2003) and Flemming and Wuertz (2019).

Degradation kinetics showed that *P. ostreatus* P5 could metabolize a mixture of phenolic acids with high efficiency because the dry weight of strain P5 mycelia increased as phenolic acids were degraded. This result was similar to those reported by other studies (Chen et al., 2011; Xie and Dai, 2015; Zhang et al., 2020). *Acinetobacter calcoaceticus* CSY-P13 from the cucumber rhizosphere effectively degraded the mixture of ferulic acid and *p*-hydroxybenzoic acid in liquid medium (Wu et al., 2018). Liu et al. (2018) reported that *Helotiales* sp. has the potential to use three phenolic acids as carbon sources and degraded them within 9 days. However, Zhou et al. (2020) found that although *Pseudomonas putida* A2 could efficiently degrade a single type of phenolic acids, a mixture of phenolic acids obviously inhibited the growth of this strain. It has been reported that microorganisms could transform one phenolic acid to another, which could be less or even more phytotoxic to plants. However, we did not detect any intermediate or transformed metabolites when phenolic acids were present in cultures of strain P5.

In this study, five phenolic compounds (*p*-hydroxybenzoic acid, vanillic acid, ferulic acid, *p*-coumaric acid, and benzoic acid) were detected from the continuous cropping soil that grew cucumber. However, Chen et al. (2011) reported six phenolic acids (*p*-hydroxybenzoic acid, vanillic acid, ferulic acid, benzoic acid, cinnamic acid, and 3-phenylpropanoic acid) in the cucumber cropping soil. These small differences might be explained by the different cucumber cultivars and natural soil microorganisms, which could degrade or convert phenolic acids (Zhou et al., 2012). This study showed that, compared with CK, total phenolic acids decreased by 35.9 and 63.2% in P5 and B2 + P5 treatments, respectively. These results suggested strain P5 can adapt to soil habitats and promote the degradation of soil phenolic acids in combination with natural microorganisms. This finding was verified by the results of Xie et al. (2017), who determined that fungal *Phomopsis liquidambari* significantly lowered the residual level of *p*-coumaric acid, vanillic acid, and *p*-hydroxybenzoic acid in soil. Wu et al. (2018, 2019) also reported that *Streptomyces canus* GLY-P2 (Wu et al., 2019) and *Acinetobacter calcoaceticus* CSY-P13 (Wu et al., 2018) could mitigate the stress of ferulic acid and *p*-hydroxybenzoic acid in cucumber by degrading them in soil.

Combining two or more agents in biocontrol is an efficient strategy for the management of soilborne pathogens and has been reported in previous studies (Awasthi et al., 2011; Yobo et al., 2011; Zaim et al., 2018; Jangir et al., 2019). To our knowledge, this study is the first report on combined application of an antagonistic *B*. *amyloliquefaciens* with a phenolic acid-degrading *P. ostreatus* for cucumber Fusarium wilt disease management. In this study, the combined application of strain B2 and P5 (B2 + P5) showed the best control efficacy of Fusarium wilt in a pot experiment (Figure 7). Similarly, several recent studies showed that combined application of *Bacillus* sp. with beneficial fungi could increase soilborne disease suppression (Zaim et al., 2018). Zaim et al. (2018) investigated the efficacy of the combination of *B. subtilis* Bs1 and *Trichoderma harzianum* T5 against *F. oxysporum* f. sp. *ciceris* in chickpeas and found more pronounced disease control in plants treated with dual inoculation. Sylla et al. (2013) also reported a high level of disease suppression on treatment with multiple strains (*B. subtilis* FZB24 and *T. harzianum* T58) against strawberry powdery mildew. This synergism can be attributed to the fact that the biocontrol agents probably use different mechanisms of biocontrol and therefore complement each other. Various biocontrol mechanisms of *Bacillus* spp. have been reported, such as antimicrobial compound production, competing for niche and nutrients, or induction of local and systemic defense responses of plant (Shafi et al., 2017; Netzker et al., 2020). We found a significant positive correlation between disease incidence and FOC abundance (Figure 9), suggesting that a decline in the pathogen population was one of the mechanisms underlying the management of plant diseases with antagonistic strain B2 inoculation. Many studies demonstrated that the accumulation of phenolic acid in cucumber continuous cropping soil is one of the key factors that resulted in serious Fusarium wilt (Ye et al., 2004; Chen et al., 2011; Jin et al., 2020). Previous studies showed that no direct antagonism was observed between strain P5 and FOC *in vitro* (Wang et al., 2020). Additionally, correlation analysis showed a positive relationship between disease incidence and phenolic acid content (Figure 9). Therefore, the prompt degradation of soil phenolic acids by strain P5 may be a major factor for reducing Fusarium wilt. Similarly, Xie et al. (2017) reported that phenolic acid-degrading fungus *Phomopsis liquidambari* significantly suppressed peanut Fusarium diseases primarily by reducing the content of phenolic acids in continuous cropping soil.

In this study, the impact of strain B2 and P5 either alone or in combination on cucumber plants grown was studied under greenhouse conditions. The results displayed that all microbial inoculant treatments positively affected shoot and root growth. Overall, the B2 + P5 treatment yielded best results over singly inoculated plants with either strain B2 or P5 (Table 3). The results of this study are in agreement with previous studies, where plants inoculated with dual inoculants, *Bacillus* spp. in combination with other plant-beneficial microbes (*Trichoderma atroviride*, *Piriformospora indica*, *Glomus mosseae*, *Penicillium bilaiae*), exhibited significantly enhanced physiological parameters when compared to singly inoculated and control plants [e.g., dry bean (Yobo et al., 2011), chickpea (Zaim et al., 2018), tomato (Anith et al., 2015), *Artemisia annua* (Awasthi et al., 2011), wheat (Hansen et al., 2020), and *Lactuca sativa* (Kohler et al., 2007)]. The enhancement of seedling growth following co-inoculation could be due to the ability of strains B2 and P5 to act through different mechanisms including producing IAA and siderophores, reducing FOC population, and enhancing the biodegradation of autotoxic phenolic acids in soil.

Several authors have suggested that combinations of introduced microbial agents have to be compatible with each other for better and more consistent disease suppression (Chien and Huang, 2020; Dugassa et al., 2021). *Bacillus* species, particularly *B. amyloliquefaciens* and *B. subtilis*, can produce a wide range of antifungal compounds such as lipopeptide antibiotics (e.g., iturin, surfactin, and fengycin) and lytic enzymes (e.g., catalase, protease, and cellulase) (Xiong et al., 2015). Kim et al. (2008) found that some *Bacillus* species inhibited mycelial growth of *P. ostreatus* on PDA plates. Whenever there is antagonism among the different microbial agents, it is not advisable to use them for mixed inoculation on plants. However, incompatibility of the inoculants could, to some extent, be circumvented by temporal/spatial separation in application (Anith et al., 2011). In this study, *P. ostreatus* P5 and *B. amyloliquefaciens* B2 were inoculated in non-rhizosphere soil before transplanting and in rhizosphere soil after transplanting, respectively, instead of inoculation with the mixture of P5 and B2. In addition, more studies are still needed to explore the interaction between these two agents, especially in the soil environment.

## Conclusion

**FIGURE 10:**
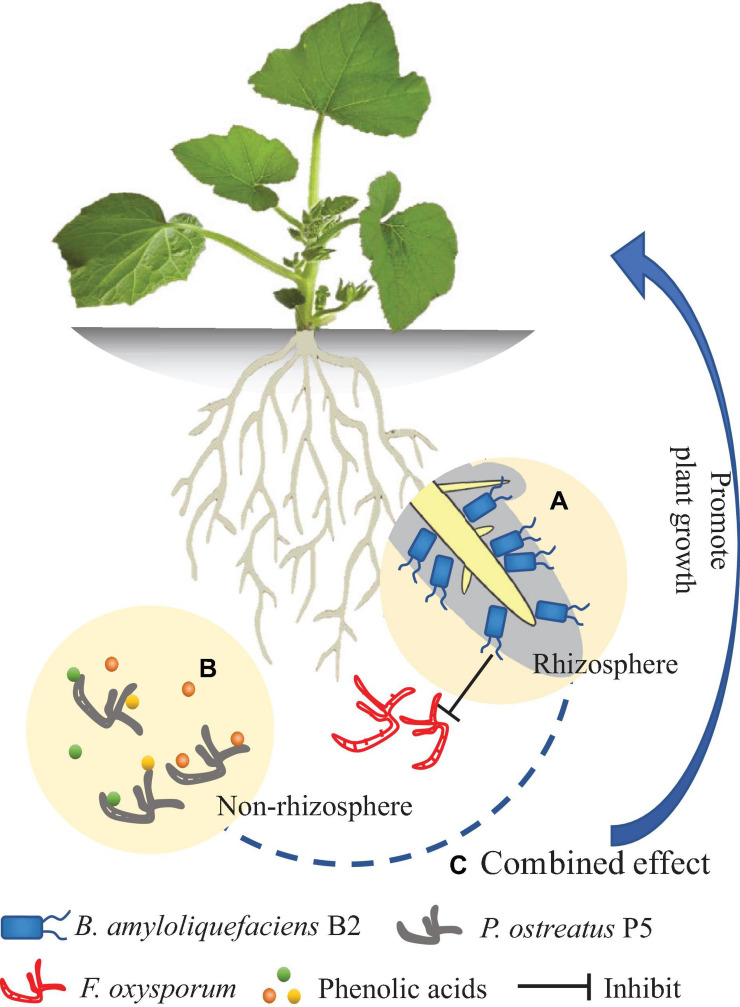
Model diagram of the effect of *Bacillus amyloliquefaciens* B2 and *Pleurotus ostreatus* P5 on plant growth. **(A)** *B. amyloliquefaciens* B2 can inhibit the *Fusarium oxysporum* in rhizosphere soil. **(B)** *P. ostreatus* P5 can biodegrade the phenolic acids in non-rhizosphere soil. **(C)** The combination of *B. amyloliquefaciens* B2 and *P. ostreatus* P5 synergistically enhances plant growth.

The new antagonist strain B2 of *B. amyloliquefaciens* exhibited several plant-beneficial traits *in vitro*. To the best of our knowledge, this study is the first to show that *P. ostreatus* can effectively degrade a mixture of allelopathic phenolic acids. The integrated treatment (B2 + P5) showed more effectiveness in suppressing Fusarium wilt disease than applying either B2 or P5 alone and significantly increased cucumber growth (Figure 10). However, as this study was performed in a pot experiment, further study is required to validate the suppressive effect of the combined application of these two agents in naturally agricultural land. The following two aspects should be considered in performing field experiments. One aspect is the effect of combined application of B2 and P5 on soil microbial community structure and function. Another is the molecular mechanisms involved in the interactions among the inoculated microbial agents, the plant and the pathogen.

## Data Availability Statement

The datasets presented in this study can be found in online repositories. The names of the repository/repositories and accession number(s) can be found below: https://www.ncbi.nlm.nih.gov/genbank/, MW308308.

## Author Contributions

HW designed the research. X-YC, MX, and FT performed the research. HW analyzed the data and wrote the manuscript. All the authors contributed to the article and approved the submitted version.

## Conflict of Interest

The authors declare that the research was conducted in the absence of any commercial or financial relationships that could be construed as a potential conflict of interest.

## Publisher’s Note

All claims expressed in this article are solely those of the authors and do not necessarily represent those of their affiliated organizations, or those of the publisher, the editors and the reviewers. Any product that may be evaluated in this article, or claim that may be made by its manufacturer, is not guaranteed or endorsed by the publisher.

## Footnotes

1
http://blast.ncbi.nlm.nih.gov/Blast.cgi
